# The protocol of a clinical quality registry for dementia and mild cognitive impairment (MCI): the Australian dementia network (ADNeT) Registry

**DOI:** 10.1186/s12877-020-01741-2

**Published:** 2020-09-07

**Authors:** Xiaoping Lin, Kasey Wallis, Stephanie A. Ward, Henry Brodaty, Perminder S. Sachdev, Sharon L. Naismith, Karolina Krysinska, John McNeil, Christopher C. Rowe, Susannah Ahern

**Affiliations:** 1 School of Public Health and Preventive Medicine, Monash University, Level 3, 553 St Kilda Rd, Melbourne, Victoria 3004 Australia; 2 Centre for Healthy Brain Ageing (CHeBA), School of Psychiatry, University of New South Wales, Sydney, New South Wales Australia; 3 Department of Geriatric Medicine, The Prince of Wales Hospital, Sydney, New South Wales Australia; 4 Dementia Centre for Research Collaboration, School of Psychiatry, University of New South Wales, Sydney, New South Wales Australia; 5 Neuropsychiatric Institute, The Prince of Wales Hospital, Sydney, New South Wales Australia; 6 School of Psychology, The University of Sydney, Sydney, New South Wales Australia; 7 Centre for Mental Health, Melbourne School of Population and Global Health, The University of Melbourne, Melbourne, Victoria Australia; 8 Department of Molecular Imaging and Therapy, Austin Health, Heidelberg, Victoria Australia; 9 Florey Department of Neuroscience and Mental Health, The University of Melbourne, Melbourne, Victoria Australia

**Keywords:** Dementia, Mild cognitive impairment, Neurocognitive disorders, Registries, Clinical quality registries, Health care quality

## Abstract

**Background:**

Dementia was identified as a priority area for the development of a Clinical Quality Registry (CQR) in Australia in 2016. The Australian Dementia Network (ADNeT) Registry is being established as part of the ADNeT initiative, with the primary objective of collecting data to monitor and enhance the quality of care and patient outcomes for people diagnosed with either dementia or Mild Cognitive Impairment (MCI). A secondary aim is to facilitate the recruitment of participants into dementia research and trials. This paper describes the Registry protocol.

**Methods:**

The ADNeT Registry is a prospective CQR of patients newly diagnosed with either dementia or MCI. Eligible patients will be identified initially from memory clinics and individual medical specialists (e.g., geriatricians, psychiatrists and neurologists) involved in the diagnosis of dementia. Participants will be recruited using either an opt-out approach or waiver of consent based on three key determinants (capacity, person responsible, and communication of diagnosis). Data will be collected from four sources: participating sites, registry participants, carers, and linkage with administrative datasets. It is anticipated that the Registry will recruit approximately 10,000 participants by the end of 2023. The ADNeT registry will be developed and implemented to comply with the national operating principles for CQRs and governed by the ADNeT Registry Steering Committee.

**Discussion:**

The ADNeT Registry will provide important data on current clinical practice in the diagnosis, treatment and care of people with dementia and MCI in Australia as well as long-term outcomes among these people. These data will help to identify variations in clinical practice and patient outcomes and reasons underlying these variations, which in turn, will inform the development of interventions to improve care and outcomes for people with dementia and MCI.

## Background

Dementia represents one of the greatest and growing challenges for health and social services across the world in the twenty-first century [1]. Dementia is a clinical syndrome characterised by irreversible, and usually progressive, impairment of cognitive functions that are of sufficient severity to impact on day to day function of the individual [2]. Mild Cognitive Impairment (MCI) is a syndrome in which cognitive impairment is evident yet daily functioning is largely intact [3]. The underlying pathological causes of dementia and MCI are manifold, with Alzheimer’s disease (AD) being the commonest pathology in late-onset cases, often in combination with other pathology such as cerebrovascular disease, Lewy Bodies or TDP-43 [1, 4].

In 2015, there were 47 million people with dementia worldwide, and the number is expected to increase to 75 million by 2030 [5]. Dementia is associated with significant medical and social care costs, with the total global cost estimated to be US$ 818 billion in 2015 [5]. In Australia, the number of people with dementia is significant and increasing. In 2019, it was estimated that there were 447,115 Australians living with dementia [6], with 250 people receiving a clinical diagnosis of dementia every day [7].

### Need for a clinical quality registry for dementia in Australia

Presently there is no disease-modifying treatment available for dementia [1, 8, 9]. Despite this, patient and carer outcomes can be improved by the provision of high-quality clinical care {Fazio, 2018 #87} [1, 8–10]. This includes a timely and supported diagnosis based on structured history taking, cognitive tests, blood screening, structural imaging, and when indicated positron emission tomography (PET) scanning or cerebrospinal fluid (CSF) analysis, as well as post-diagnostic medical and psycho-social care which is tailored to meet unique individual needs of people with dementia and incorporates support for family carers [1, 8–11]. In addition, it is recommended to engage persons with dementia and their family carers in the planning for the future, including consideration of end of life care [1, 8–11].

However, the diagnosis and care of persons with dementia is varied and inconsistent across Australia [11–13]. Dementia diagnosis and initial management may occur in various settings, including general practice, hospitals, multidisciplinary memory clinics, private specialist practices, with the potential for significant variation in service accessibility, diagnostic processes, and effectiveness in dementia care within and across these different settings [12, 13]. Post-diagnostic care in Australia is usually co-ordinated in primary care, with variable access to specialised medical and multidisciplinary care, and only some memory clinics offering longer-term follow-up [12, 13].

In addition, there is clear evidence that people with dementia often have poorer long-term outcomes compared to those without dementia. For example, people with dementia are more likely to be admitted to hospitals and are at a higher risk of in-hospital death, institutionalisation at discharge, longer lengths of stay, as well as intermediate negative outcomes, such as falls, hospital-acquired infection or hospital acquired functional decline [14, 15]. However, currently, there is no systematic way to measure the variation in the care provided to people with dementia or to monitor long-term outcomes among this group in Australia, which is of vital importance to drive improvements in patient and carer outcomes.

A clinical quality registry (CQR) for dementia can address this gap by providing information on processes and outcomes of clinical care provided to people with dementia. CQRs are organisations which “systematically monitor the quality (appropriateness and effectiveness) of health care, within specific clinical domains, by routinely collecting, analysing and reporting health-related information” [16]. Importantly, CQRs have proven to be one of the most clinically valued tools to reduce variation in clinical practice and improve quality of care, based on evidence of CQRs of patients undergoing different surgeries (e.g., cardiac surgery and dialysis and transplant) and with different diseases (e.g., stroke and different types of cancer) [17–20]. Specifically, CQRs can improve quality of care by giving clinicians and health services information about how their outcomes benchmark with others, both locally and, where appropriate, internationally [16]. For health services and government agencies, CQRs can provide information to measure and monitor quality of care, in particular, to detect aspects of care that significantly deviate from standards and to identify variations in clinical practice and outcomes, at both a local and jurisdictional level [16]. CQRs can also facilitate recruitment of patients into research, and when new therapeutic agents become available, monitor uptake and long-term outcomes.

Dementia was identified as a priority area for development of a CQR in 2016 by the Australian Commission for Safety and Quality in Heath Care (ACSQHC), based on the high burden of disease, significant consequences of poor-quality care and support from relevant clinical and consumer organisations [21]. Several dementia CQRs have been established internationally, such as the Swedish Dementia Registry (SveDem), Norwegian Dementia Registry (NorKog), and the Danish Dementia Registry [22]. There is evidence that these dementia CQRs “facilitate better diagnosis, management, and care of people with dementia, as well as caregiver support, across the course of the illness” and have great potential to “reduce cost of dementia and improve standards of diagnosis and care” [22].

### The Australian dementia network (ADNeT) Registry

**Fig. 1:**
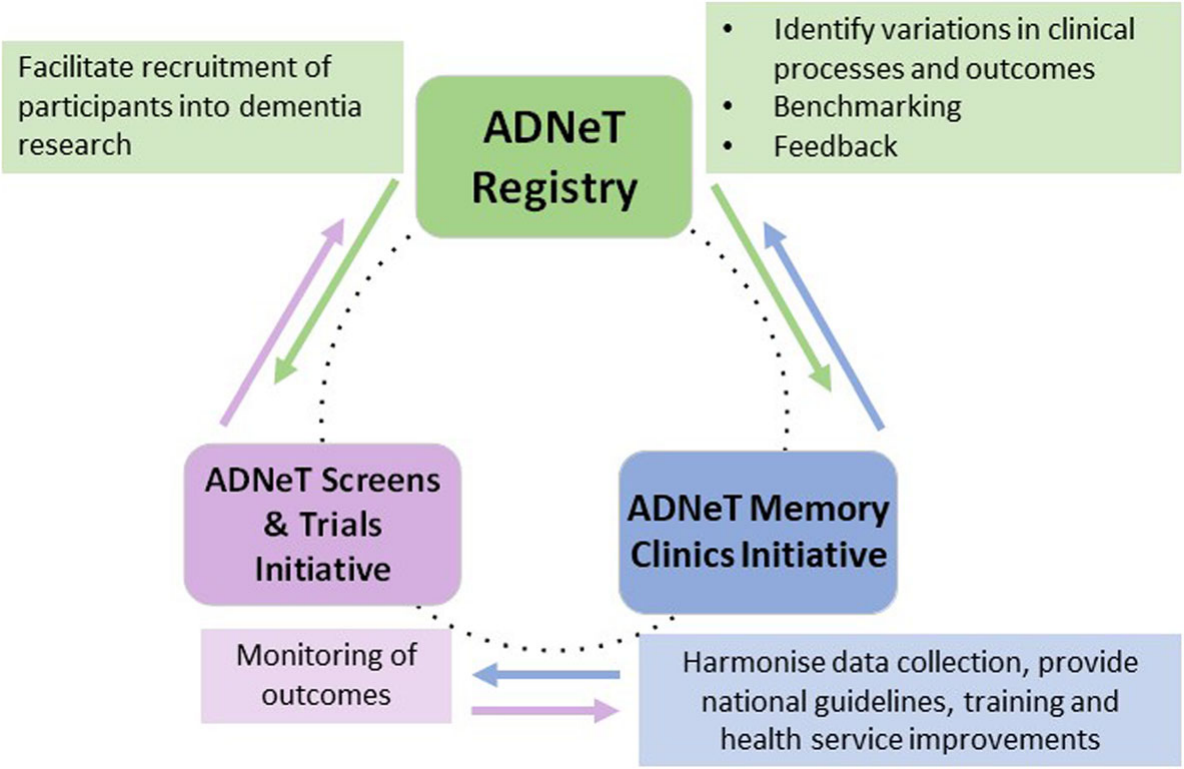
Three key pillars within the Australian Dementia Network (ADNeT) initiative

The Australian Dementia Network (ADNeT) Registry is being established as part of the ADNeT initiative, with funding from the National Health and Medical Research Council (NHMRC) National Institute for Dementia Research (NNIDR) program. ADNeT is a multi-institutional consortium of dementia researchers and clinicians across Australia and represents a comprehensive, integrated and coordinated approach to dementia research and clinical practice improvement. There are three key pillars within the ADNeT initiative: 1) the ADNeT registry, which will establish a CQR for people with dementia and MCI, 2) the ADNeT Memory Clinics Initiative, which will establish a national network of memory clinics across Australia and support quality improvements in memory clinics, and 3) the ADNeT Screens and Trials Initiative, which will develop state-of-the-art dementia clinical trial sites across Australia and identify and recruit a cohort of participants suitable to engage in dementia clinical trials. Whilst these are three distinct components, it is anticipated that they will work synergistically to improve dementia research and clinical practice in Australia (see Fig. 1).

The primary aim of the ADNeT Registry is to collect data to monitor and enhance the quality of care and patient outcomes for people diagnosed with either dementia or MCI in Australia. The secondary aims of the ADNeT Registry are to facilitate the recruitment of participants into dementia research and trials, especially the ADNeT Screens and Trials Initiative, and to establish a resource to facilitate further study into the risk factors for, and trajectory of, dementia and MCI in Australia. The ultimate vision of the ADNeT Registry is to eventually expand to all diagnostic settings and services for dementia in Australia, and register the entire population of persons newly diagnosed with either dementia or MCI, and in doing so, systematically drive improvements in the quality of care and patient outcomes. This paper describes the protocol for establishing the ADNeT registry.

## Methods

### Design and participants

The ADNeT Registry is a prospective CQR of patients newly diagnosed with either dementia or MCI. Inclusion criteria for participants are 1) new clinical diagnosis of either dementia or MCI from participating sites within Australia, and 2) aged 18 years and over. The exclusion criterion is patients who are not permanent residents of Australia.

### Process to establish the ADNeT registry

The methodologies for implementation of the ADNeT registry will initially undergo pilot testing at several sites. Learnings from the pilot stage will inform necessary amendments and inform the national roll-out of the ADNeT Registry. To inform the establishment of the Registry, a Modified Delphi Study was conducted to inform the development of Clinical Quality Indicators (CQIs) for the Registry. CQIs are specifically defined and measurable items which provide an indication of quality of care [23]. Through the Modified Delphi study, a set of CQIs was proposed for the ADNeT Registry (See [24] for details). These CQIs capture quality of care and patient outcomes across the trajectory of care for people with dementia and MCI, and cover five areas: diagnosis, management, access to services and support, potentially preventable complications, and disease progression. These CQIs have been presented to various stakeholders (including people with dementia and MCI, carers, clinicians, service providers, and government bodies) for feedback at various meetings and are being reviewed by the ADNeT Registry Steering Committee.

Following this Modified Delphi Study, the ADNeT registry team developed recruitment, consent, and data collection methods which are reported in this protocol paper. A Minimum Data Set (MDS, see Table 1 for key data elements), a data dictionary and relevant patient-facing documents were also developed. The ADNeT Registry project was approved by the Alfred Hospital Human Research Ethics Committee (HREC) under the National Mutual Acceptance Scheme (Project Number: 44037, Approval date: 27/08/2018).

**Table 1:** Key data elements in ADNeT Registry Minimum Data Set

Category	Key data elements
Patient identifiers	First name, Last name, Sex, Date of birth
Patient demographic information	Country of birth, Preferred spoken language, Aboriginal and/or Torres Strait Islander status, Highest level of education, Living arrangements, Residential setting
Information relevant to recruitment methods	Capacity to opt out, Person responsible (if appropriate), Communication of diagnosis
Baseline clinical data	Referral date, Initial appointment date, date of dementia or MCI diagnosis, Type of dementia or MCI, Scores of completed cognitive assessments (e.g., MMSE, RUDAS, MoCA, etc) Core blood tests undertaken within the 12 months prior to or at diagnosis (Yes/No), Structural neuro-imaging completed within the 12 months prior to or at diagnosis (e.g., CT, MRI) completed (Yes/No), Functional neuro-imaging completed undertaken within the 12 months prior to or at diagnosis (e.g., PET, SPECT) (Yes/No), Cholinesterase inhibitor recommended or prescribed (Yes/No), Total of prescribed medication, Comorbidity (e.g., history of stroke, hypertension, etc), Independent in personal activities of daily living (Yes/No), Independent in instrumental activities of daily living (Yes/No)

*MMSE* Mini Mental State Examination, *RUDAS* Rowland Universal Dementia Assessment Scale, *MoCA* Montreal Cognitive Assessment, *CT* Computerized tomography, *MRI* Magnetic resonance imaging, *PET* Positron emission tomography, *SPECT* Single-photon emission computed tomography

The Alfred Hospital HREC required a legal opinion be included in the ethics application of multi-state studies involving participants unable to provide informed consent to confirm that the recruitment, consent processes and associated documentation comply with the relevant legislation in each of the States and Territories involved. As such, we underwent extensive legal consultation when developing the recruitment and consent methods reported here.

### Participating sites

Participating sites in the ADNeT registry initially include memory clinics and individual medical specialists (e.g., geriatricians, psychiatrists and neurologists) involved in the diagnosis of dementia. Memory clinics in Australia function mainly as diagnostic clinics, although some offer ongoing assessment and care co-ordination [12, 13]. Persons with MCI are recommended to have a reassessment of cognition within 18 months post diagnosis to monitor changes in cognitive functioning [1, 8–11].

In Australia, dementia diagnosis also takes place in other settings such as general practice, hospital inpatient wards, residential aged care facilities, and relevant community services (e.g., community aged care assessment teams) [12]. We are conducting sub-studies to explores feasibility of recruiting patients from these settings to increase the coverage of the ADNeT Registry, as well as entry of “prevalent” cases of dementia via relevant datasets (e.g., aged care assessment datasets).

Site participation in the ADNeT Registry is voluntary. Ethics and/or governance authorisation are/is sought before the commencement of participant recruitment at each site as required.

### Recruitment and consent methods

**Fig. 2:**
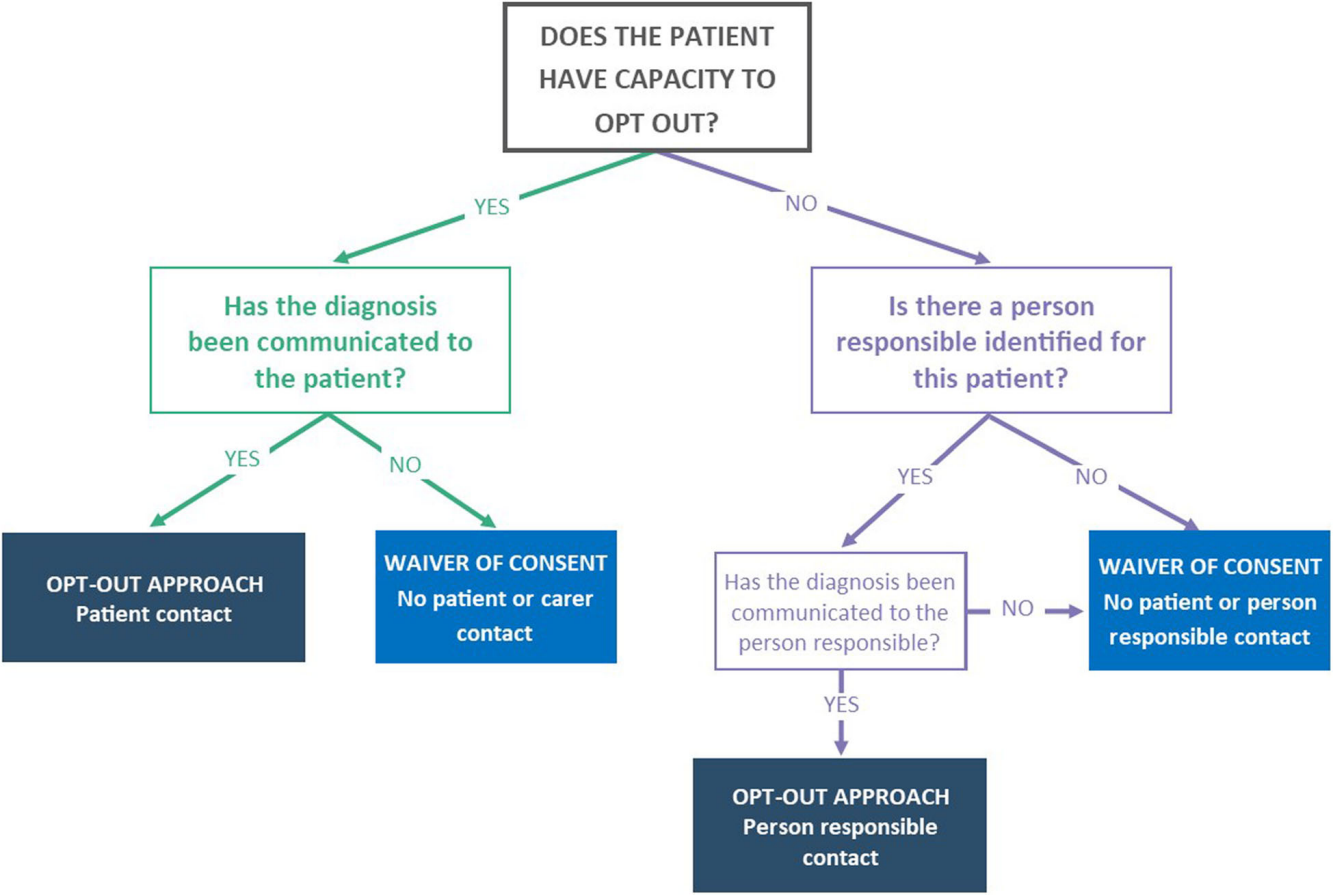
ADNeT Registry recruitment methods and three determinants

Participants will be recruited using either an opt-out approach or waiver of consent based on three key determinants using information from patients’ medical records, including:

1. Whether or not the patient has capacity to be involved in the opt-out process,
2. If the patient does not have capacity, whether or not a person responsible has been identified for the patient, and
3. Whether or not the diagnosis has been communicated to the patient (for those having capacity to opt out) and the person responsible (for those not having capacity to opt out) (see Fig. 2).

Capacity to be involved in the opt-out process is defined in the ADNeT registry as having capacity to understand the information relevant to the decision for remaining in or opting out of the ADNeT Registry, use this information to make a participation decision, and communicate participation decision. This definition is developed based on relevant Australian federal and state legislation (e.g., [25]) and the literature (e.g., [26]). Clinicians working in participating sites will be required to assess patients’ capacity in these three areas and record this in medical records.

#### The opt-out approach

The opt-out approach, which means that participants will be included in the ADNeT Registry unless they notify the ADNeT Registry that they would not like to participate, will be used for two patient groups: 1) those who have capacity to opt out and have been informed of their diagnosis, and 2) those who do not have capacity to be involved in the opt out approach and have an identified person responsible with whom the diagnosis has been communicated. The opt-out approach has been used as a recruitment approach in most CQRs in Australia and maximises the coverage rate of CQRs among their target populations, thus increasing clinical validity and ensuring meaningful comparison of variation in health outcomes across sites [27]. In turn, it increases the capacity of CQRs to inform and influence clinical guidelines, policy development and the public health agenda [28, 29].

Using an opt-out approach, participants will be recruited using three steps:

1. Clinicians provide potential participants or their persons responsible (where appropriate) with an ADNeT Registry Introductory Postcard,
2. Site coordinators identify eligible patients and enter their details into the ADNeT Holding Database,
3. ADNeT registry staff access ADNeT Holding Database and contact newly-identified patients or person responsible to inform them of the Registry and the various opt-out methods. In addition, patients and persons responsible who are also recognised as carers for the patients, will be invited to complete questionnaires about their experience of living with dementia and MCI or caring for people with dementia and MCI, respectively.

A four-week opt-out period will be provided for patients. During this time, participants or persons responsible will be able to opt out using email, toll free phone and reply-paid post. Participants and persons responsible can also opt out anytime post this four-week period.

#### Waiver of consent

The registry will use waivers of consent to recruit four groups of patients: 1) patients who have capacity but the diagnosis has not been communicated to the patients, 2) patients who do not have capacity to opt out nor an identified person responsible, 3) patients who do not have capacity and the diagnosis has not been communicated to the person responsible, and 4) patients who die prior to the recruitment period. For these patient groups, there will be no patient or person responsible contact and the patients will be automatically recruited into the ADNeT Registry. Where a patient is recruited utilising a waiver of consent, the site will not collect patient, person responsible or carer contact details. However, a number of key patient identifiers are collected to enable longitudinal data collection via data linkage.

#### Current recruitment progress

Participant recruitment and data collection for the ADNeT Registry commenced in February 2020. The Registry will approach all specialised dementia diagnostic services and memory clinics in Australia nationwide, representing about 50 clinics, by the end of 2023. In Australia, it is estimated that there are approximately 86,438 new (incident) cases of dementia a year [7]. Whilst systematic data on diagnostic locations is limited, one small Australian study conducted with patients with dementia in a sub-acute hospital found that 15% of prevalent dementia diagnoses had been made in memory clinics [30]. As such, we anticipate that by the end of 2023, the Registry will recruit at least 10,000 participants. The Registry will also be expanded to encompass other diagnostic settings, such as private specialists and general practice, similar to the expansion of the SveDem. As a clinical quality registry, we anticipate ongoing participant recruitment for the Registry.

##### Data collection

The ADNeT registry will collect information from four sources: participating sites, registry participants (except those recruited under waiver of consent), their carers (except those recruited under waiver of consent), and linkage with administrative datasets (see Table 2). Data collected at baseline from the participating sites will be based on the MDS and a data dictionary has been developed to ensure consistency in data collection. The Registry also intends to collect follow-up clinical data from participating sites and will explore collection methods and timepoints for follow-up clinical data.

**Table 2:** Proposed data sources, data content and data collection time points for ADNeT Registry

Data sources	Data content	Data collection time points		
		At recruitment	Clinical follow-up appointments	Anticipated annual patient and carer follow-ups
Participating sites	Patient identifiers (e.g., first name, last name, and date of birth)	x		
	Patient demographic information (e.g., country of birth, and highest attained level of education)	x		
	Information relevant to recruitment methods (e.g., capacity to opt out, communication of diagnosis)	x	x	
	Baseline clinical data (e.g., date of diagnosis, type of dementia)	x		
	Follow-up clinical data		x	
Registry participants (except those recruited under waiver of consent)	Patient survey	x		x
Carers (except patients recruited under waiver of consent)	Carer survey	x		x
Administrative datasets (via data linkage)	Anticipated to include data routinely collected by various government bodies, such mortality, hospitalisation, prescribed medication, and aged care service utilisation			Periodically as appropriate

The registry intends to collect data from registry participants and their carers (except those recruited under waiver of consent) via self-completed patient and carer surveys. Internationally, patient-reported outcome measures (PROMs) and patient-reported experience measures (PREMs) are increasingly being used to provide patients’ perspectives in the assessment of health care quality and service improvement [31, 32]. As stated in a literature review commissioned by the Australian Commission on Safety and Quality in Health Care [31], PROMs and PREMs are “integral parts of a movement towards patient-centred systems of structuring, monitoring, delivering and financing health care. Increasingly, quality is being seen as defined by the patient, not just by the clinician or policymaker” (p.4). Currently, no PROM and PREM has been developed in the context of a dementia registry. We are conducting a sub-study to develop patient surveys for the ANDeT Registry based on the recent development of PROMs and PREMs.

In addition, carers play an important role in supporting people with dementia, and this can have significant impacts, both positive and negative, on their own well-being. As such we intend to develop carer surveys to collect information on carer-reported outcome and experience. We anticipate using the 12-item Zarit Burden Interview [33] as part of the initial carer survey to measure carer outcome based on the level of carer burden.

The registry plans to collect data from participants and their carers at the time of recruitment and annually (anticipated) to track changes in patient- and carer-reported outcomes and experience as dementia progresses. Because there are likely declines in participants’ cognitive functions, which will have implications on their ability to provide consent, a sub-study is being conducted to explore appropriate follow-up methods and timepoints for people with dementia and MCI who have capacity to opt out at the time of recruitment.

Finally, the ADNeT Registry will collect information through linkage with data routinely collected by various government bodies, such as mortality, hospitalisation, prescribed medication, and aged care service utilisation. The purpose of the data linkage is to provide a comprehensive and longitudinal picture of outcomes among people with dementia and MCI. It is anticipated that the Registry will apply for data linkage (with a waiver of consent) when there are sufficient number of participants within the Registry.

### Data management

The ADNeT Registry will use a secure, web-based platform to enter and manage data. All users of the ADNeT databases need to log in to the databases through a login screen with a pre-configured username and password controlled by administrators of the system. These databases are housed and managed in an International Organization for Standardization (ISO) 27,001 certified environment.

The ADNeT Registry will implement a number of strategies to ensure the quality, consistency and interpretability of data recorded. This will include strategies for data entry (such as in-built logic checks to ensure data meets formatting and value requirements and validity), as well as quality assurance processes post data entry (such as routine cleaning and quality checks of data received in the Holding Database). There will also be periodic feedback, such as data completeness and quality reports, will be provided to participating sites to ensure improved consistency and quality of data collected. Training, education and ongoing liaison with participating sites will be provided to supporting high-quality data collection.

### Data analysis

Initial data analysis for the ADNeT Registry will focus on descriptive analysis to provide aggregate summary information regarding cohort characteristics, CQIs, and patient and carer reported measures when available. These data will provide the basis for reporting and feedback to participating sites as well as annual public reports. The registry will also conduct analyses to provide performance metrics such as opt-out rate, cumulative recruitment of participating sites, and response rates for patient and carer reported measures (when available).

When participant volume is sufficient, data regarding CQIs will be risk-adjusted and benchmarked. Crude and risk-adjusted funnel plots will be produced for quality indicators as appropriate. Data linkage will be conducted periodically and analyses will be undertaken to provide a comprehensive and longitudinal picture of patient outcomes.

Importantly, the ADNeT Registry has in-built exporting functionality to enable data extraction by participating sites. This is key because the Registry’s primary purpose is to help participating clinicians review their clinical practice, and use this information to drive service improvements. During the pilot stage we will engage pilot sites to refine reporting functionality for participating sites. The Registry Staff will also provide participating sites with site reports on a regular basis. These reports will provide information on participant characteristics and CQIs (risk adjusted and benchmarked if appropriate). In addition, these data will be used by the ADNeT Memory Clinics Initiative, which will establish a national network of memory clinics across Australia, to inform the development of training models, national guidelines, and quality improvement activities in memory clinics.

### Governance

The registry will be developed and implemented to comply with the national operating principles for CQRs as set out by the ACSQHC [16]. The project will be managed by the ADNeT Registry Steering Committee which will provide governance oversight, strategic direction and ensure that agreed policies and procedures are adhered to. The Steering Committee comprises key clinician craft group representative (geriatric medicine, psychiatry, neurology, general practice, nursing, and neuropsychology), data custodian representatives, ADNeT investigators, dementia-related peak bodies, and representatives of people with dementia and/or MCI and their carers.

## Discussion

In light of the projected increase in prevalence of dementia and MCI in Australia and great variance in care practices, the ADNeT Registry is being established as a CQR to systematically collect data to monitor the quality of care and patient outcomes among people newly diagnosed with either dementia or MCI. The ADNeT Registry will provide important data on current clinical practice in the diagnosis, management and treatment of people with dementia and MCI in Australia and long-term outcomes among these people. These data will help to identify variations in clinical practice and patient outcomes and reasons underlying the variations, which in turn, will inform the development of interventions to improve care and outcomes for people with dementia and MCI.

There are a number of strengths in the design of the ADNeT Registry. First, the ADNeT registry is operated as part of the wider ADNeT initiative, which is a multi-institutional consortium of dementia researchers and clinicians across Australia. Embedding the ADNeT Registry within the wider ADNeT initiative brings a number of benefits. Specifically, the ADNeT initiative includes the ADNeT Memory Clinics which seeks to establish a national network of memory clinics across Australia and support quality health service improvements, data harmonisation as well as training and national guidelines. The ADNeT Registry will identify variations in clinical practice and patient outcomes among participating sites, that can be explored and supported through the Memory Clinic network. Conversely, Memory Clinics will enable local quality improvement activities and staff professional development activities, the effectiveness of which can be monitored via the ADNeT Registry. Additionally, the ADNeT Registry will provide a mechanism to facilitate recruitment of participants to clinical trials of emerging treatments. Should a disease modifying agent for dementia be identified, the ADNeT Registry will provide a mechanism for monitoring the uptake and real-world long-term outcomes for any new treatment.

Second, two different recruitment and consent methods (i.e., an opt-out approach and waiver of consent) have been developed based on three key determinants (i.e., capacity to be involved in the opt-out process, person responsible and communication of diagnosis) to ensure that the ADNeT Registry reaches maximum coverage, while respecting patients’ choice and privacy. For example, there has been considerable debate regarding the ethical and practical issues surrounding disclosing a dementia diagnosis [34, 35]. While most ethical guidelines promote disclosure of a diagnosis of dementia to the patient, some clinicians might choose not to for reasons such as patients requesting not to be informed of the diagnosis, concerns about impaired insight among patients, concerns about risk to patients’ psychological well-being or requests from family. We have taken this variation in clinical practice into consideration and will use a waiver of consent model to recruit patients or persons responsible who are not informed of the dementia diagnosis. Compared to other dementia CQRs which typically uses only one consent method (e.g., an opt-out approach for the SveDem and informed consent for NorKog), using two consent models enables the ADNeT Registry to include a larger group of people with dementia and MCI and maximise the registry coverage and inclusiveness. Additionally, including people who are not informed of their diagnosis will provide information on diagnosis disclosure practices and factors that might contribute to this practice.

Third, the ADNeT Registry will collect information from various sources, including participating sites, registry participants and their carers, and data linkage, and follow up patients and carers beyond the diagnosis stage. This will enable a more comprehensive and longitudinal picture of care and patient outcomes among people diagnosed with dementia and MCI. Importantly, the patient and care surveys in the ADNeT Registry will incorporate patient- and carer-reported outcome and experience measures in its data collection. Internationally, PROMs and PREMs are increasingly being used to provide patients’ perspectives in the assessment of health care quality and service improvement [31, 32] and reflects “a movement towards patient-centred systems of structuring, monitoring, delivering and financing health care” [31]. Currently, we are not aware of any dementia CQRs that routinely collect patient- and carer-reported outcome and experience data, so their proposed inclusion in the ADNeT registry represents an innovation to incorporate patient and carer perspectives when measuring the quality of care and patient outcomes among people with dementia and MCI.

We are also conducting a number of other sub-studies to maximise the potential of the ADNeT Registry. These include studies to explore the feasibility of recruiting patients from other settings, such as hospital inpatient wards and general practice to maximise the coverage of the ADNeT Registry and following up patients through General Practitioners (GPs) and residential care services and via linkage with other relevant registries, such as the Australian and New Zealand Hip Fracture Registry [36] and the Australian Stroke Clinical Registry [37], to provide longitudinal clinical data. Particularly, the recent mandatory requirement in Australia for residential care homes to collect minimum quality indicator information on its residents will provide further quality of care information for persons with dementia towards the end of their lives [38], adding to the baseline clinical and early follow up information in the ADNeT Registry. In addition, we are exploring the feasibility of expanding the registry by recruiting “prevalent” cases of dementia from relevant data sources in Australia, such as via the Registry of Older South Australians (ROSA), which recruits persons at the time of an aged care assessment in South Australia [39]. The applicability of such a model to supplement the ADNeT Registry data collection is being evaluated concurrently during the pilot stage. Such initiatives have the potential to provide comprehensive outcome information for persons with dementia across multiple settings and multiple stages of life, thus maximising the coverage, and thus utility of the Registry data.

In conclusion, the ADNeT Registry is a CQR for people newly diagnosed with either dementia or MCI in Australia and has great potential to improve the quality of care and patient outcomes for this significant and vulnerable population in Australia.

## Data Availability

Not applicable.

## Abbreviations

ADNeT: Australian Dementia Network
ACSQHC: Australian Commission for Safety and Quality in Heath Care
CSF: Cerebrospinal fluid
CQIs: Clinical Quality Indicators
CQR: Clinical Quality Registry
HREC: Human Research Ethics Committee
ISO: International Organization for Standardization
MCI: Mild Cognitive Impairment
MDS: Minimum Data Set
NHMRC: National Health and Medical Research Council
NNIDR: NHMRC National Institute for Dementia Research
NorKog: Norwegian Dementia Registry)
PET: Positron emission tomography
PREMs: Patient reported experience measures
PROMs: Patient reported outcome measures
SveDem: Swedish Dementia Registry
